# The nearly complete mitochondrial genome of the door snail *Euphaedusa aculus* (Stylommatophora: Clausiliidae) and phylogenetic analysis

**DOI:** 10.1080/23802359.2024.2427105

**Published:** 2024-11-20

**Authors:** Qi-Chao Liang, Ke-Xin Tao, Dong-Dong Qiao, Pei Wang, Hao-Fei Yin, Qian-Qian Yang

**Affiliations:** a Zhejiang Provincial Key Laboratory of Biometrology and Inspection & Quarantine, College of Life Sciences, China Jiliang University, Hangzhou, China; b National Wetland Museum of China, Hangzhou, China; c Key Laboratory of Molluscan Quarantine and Identification of GACC, Fujian, China

**Keywords:** Mitogenome, Clausiliidae, door snail, phylogenetic relationships

## Abstract

The family Clausiliidae was proposed as a model for studying the diversity of breeding biology in land snails. This study assembled a nearly complete mitogenome (15,417 bp) for the clausiliid snail *Euphaedusa aculus*. The mitogenome includes 13 protein-coding genes (PCGs), two ribosomal RNAs, and 22 transfer RNAs, with a gene order consistent with other clausiliids. Most PCGs use ATN start codons and complete stop codons, except for *atp6, nad4*, and *nad5*, which start with TTG, and *atp6, cox3*, and *nad3*, which have incomplete stop codons. Phylogenetic analyses revealed that *E. aculus* is closely related to *E. planostriata*. This mitogenome provides a valuable molecular resource for future evolutionary research on land snails.

## 1. Introduction

*Euphaedusa aculus* (W. H. Benson, 1842) is a terrestrial snail from the family Clausiliidae (Gastropoda: Stylommatophora), commonly known as door snails. Clausiliidae is probably the largest family of land snails with about 1300 species, commonly habitat in rocky mountainous regions, damp rocks, walls, and tree trunks across Europe, Asia, and South America (Rosenberg 2014, Chen and Zhang 1999). The clausiliids have been proposed as a model taxa for investigating the diversity of breeding biology among land snails, due to many species being viviparous and embryo-retention reproductive (Sulikowska-Drozd et al. 2020). *E aculus* is typically viviparous, with up to 12 embryos in shell and hosting the greatest number of embryos among the clausiliid clade (Sulikowska-Drozd et al. 2020). Due to their distinctive clausilial apparatus which is believed correlated with the reproductive strategy, the taxonomy of Clausiliidae attracted much attention (Sulikowska-Drozd et al. 2014). Uit de Weerd and Gittenberger (2013) conducted a phylogenetic analysis of 67 Clausilidae species using 28S rRNA and histone H3 and H4 and divided into seven subfamilies. Mamos et al. (2021) reconstructed the evolution of reproductive strategies using time-calibrated molecular phylogenetics, reproductive mode examinations and ancestral state reconstruction in the subfamily Phaedusinae and found one non-viviparous Southeast Asian clausaliid clade species (or species clade). Hausdorf and Neiber (2022) reconstructed the phylogenetic trees of almost all existing species from the tribe Clausiliini using mitochondrial and nuclear DNA sequences and confirmed the validity of seven genera.

Currently, only two species from the family Clausiliidae have complete mitochondrial genomes available in the public database, i.e. *Albinaria caerulea* and *Euphaedusa planostriata* under GenBank accession number NC001761 and MW118059, respectively. Here, we assembled the nearly complete mitochondrial genome of *E. aculus* to provide useful data for studying the molecular phylogeny and evolutionary features of this group.

## 2. Materials and methods

### 2.1. Sample collection and preservation

**Figure 1.**
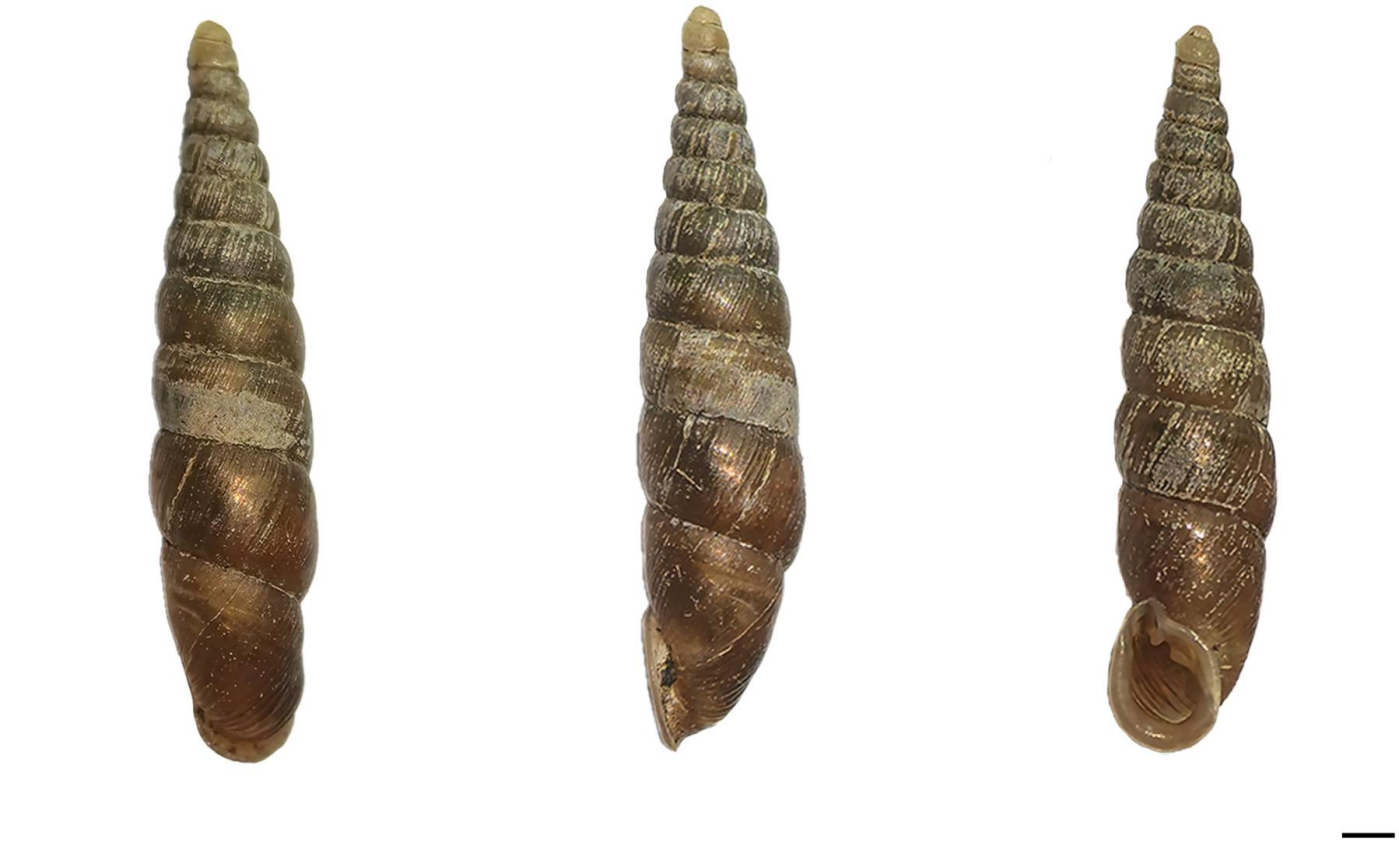
The shell morphology of *Euphaedusa aculus*. Scale bar = 1 mm. This is an original image by the authors.

The samples (Figure 1) used in this study were collected from Xixi National Wetland Park, Hangzhou, Zhejiang, China (30°16′14″N, 120°03′45″E). We identified the specimens as *E. aculus* based on morphological characteristics and the COI barcode fragments. We stored all the specimens in 100% ethanol at −20 °C and deposited at the Zhejiang Provincial Key Laboratory of Biometrology and Inspection & Quarantine, China Jiliang University, Zhejiang, China (Qian-Qian Yang, yqq@cjlu.edu.cn) under the voucher number Eacu201601.

### 2.2. DNA extraction, sequencing, and annotation

We extracted the genomic DNA from the soft tissues of an individual using the TIANamp Genomic DNA kit (TIANGEN, Beijing, China) following the manufacturer’s instructions. We characterized the genomic DNA using the Nanodrop 2000 spectrometer (Thermo Fish Scientific, Wilmington, DE, USA), and then the DNA sample was sent to LC-Biotechnologies, Co., Ltd. (Hangzhou, China) to construct a library and sequence using the Illumina MiSeq platform with a MiSeq Reagent Kit v3 (Illumina, San Diego, CA). After quality control, we obtained a total of 18,529,889 clean paired-end reads with a read length of 150 bp in FASTQ format. We assembled the sequence with Geneious v11.1.1 (https://www.geneious.com) by taking a partial sequence of *cox1* as the initial reference. The contigs were extended using the assembly parameters of the minimum overlap of 30 bp and minimum overlap similarity of 100%.

We used the MITOS web server (Bernt et al. 2013) to annotate the mitochondrial genes. We manually checked the start and stop codons of protein-coding genes (PCGs) using ORF Finder in Geneious v.11.1.4 (https://www.geneious.com) and then used BLASTP searches in NCBI (https://www.ncbi.nlm.nih.gov/). The circular graph of the mitogenome was drawn by CGView (Grant et al. 2023).

### 2.3. Phylogenetic analysis

We utilized a total of 13 complete mitogenomes from the order Stylommatophora to reconstruct phylogenetic relationships, including *Albinaria caerulea* and *Euphaedusa planostriata* from the family Clausiliidae. Additionally, *Melampus sincaporensis* from the order Ellobiida was employed as an outgroup. We aligned the nucleotide sequences of the 13 PCGs and two rRNAs individually by ClustalW multiple alignments implemented in the Alignment Explorer program with the default parameters implemented in MEGA X (Kumar et al. 2018). Then, we removed ambiguous regions in the alignment of each gene using Gblocks Server v0.91b (Castresana 2000, Dereeper et al. 2008, Dereeper et al. 2010) under a relaxed strategy. Finally, we concatenated all the gene alignments and obtained a sequence matrix of 1,0178 bp. We reconstructed phylogenetic trees using the maximum likelihood (ML) and Bayesian inference (BI) approaches using IQtree web server (http://iqtree.cibiv.univie.ac.at/) (Trifinopoulos et al. 2016) and MrBayes (v3.2.7a) (Ronquist et al. 2012). We reconstructed the ML method with 1000 bootstrap repeats. For the BI analysis, we conducted two independent runs with four Markov chains for 2,000,000 generations, estimated majority-rule consensus trees by combining results from the duplicate analyses, and sampled Markov chains every 1000 generations discarding the first 25% as burn-in. When the average standard deviation of split frequencies was below 0.01, the stationarity was considered to have been reached, and the analysis was stopped. The tree was visualized using FigTree (v1.4.4).

## 3. Results

**Figure 2.**
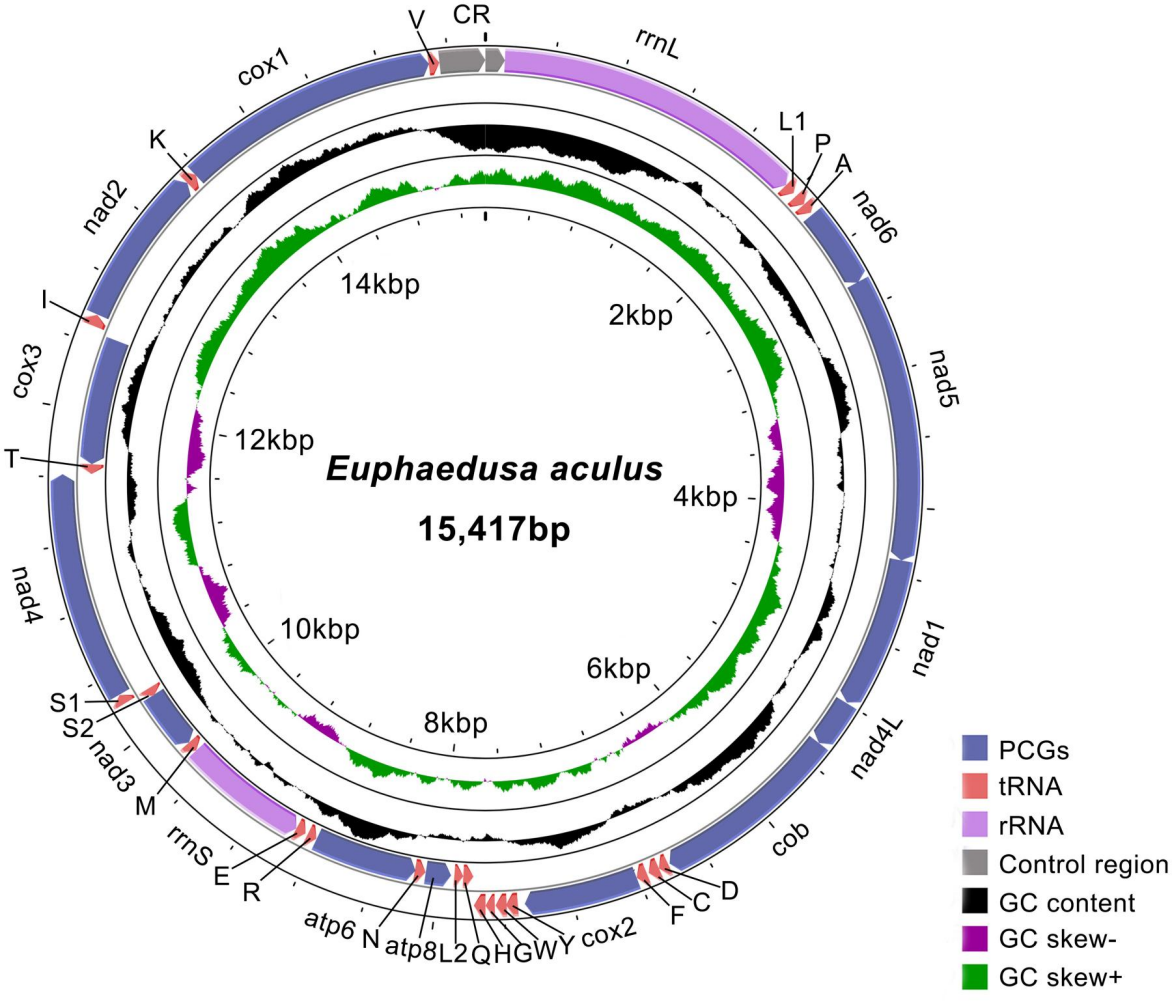
Mitogenome map of *Euphaedusa aculus*. Arrows indicate the orientation of gene transcription. The protein-coding genes (PCGs) are shown as blue arrows, rRNA genes as purple arrows, and tRNA genes as pink color arrows. The GC content is plotted using a black sliding window as the deviation from the average GC content of the entire sequence. The GC-skew is plotted using a colored sliding window (green and orchid color) as the deviation from the average GC skew of the whole sequence. Abbreviations of gene names are: *atp6* and *atp8* for ATP synthase subunits 6 and 8, *cox1-3* for cytochrome oxidase subunits 1-3, *cytb* for cytochrome b, *nad1-6* and *nad4L* for NADH dehydrogenase subunits 1-6 and 4 L, *rrnL,* and *rrnS* for large and small rRNA subunits. tRNA genes are indicated with one-letter corresponding amino acids; the two tRNA genes for leucine and serine have different anticodons.

We obtained a nearly complete mitochondrial chromosome of *E. aculus* with 15,417 bp in length with an average read coverage depth of 192× (Figure S1). We have deposited the sequence in GenBank under the accession number PP485172. We failed to assemble the complete control region due to the difficulty affected by the tandem duplication. The mitogenome of *E. aculus* encodes 13 PCGs, two rRNA, and 22 tRNA genes, which exhibited strong AT bias with a base composition of 32.0% A, 38.4% T, 13.9% C, and 15.7% G. All the PCGs had an ATN start codon except for a*tp6*, *nad4*, *and nad5*, which start with TTG. All the PCGs had a complete stop codon TAA or TAG, while *atp6* had an incomplete stop codon TA and *cox3* and *nad3* have an incomplete stop codon T. The lengths of the tRNAs ranged from 54 to 69 bp. Small and large rRNA genes are 794 bp and 1826 bp, respectively (Figure 2).

**Figure 3.**
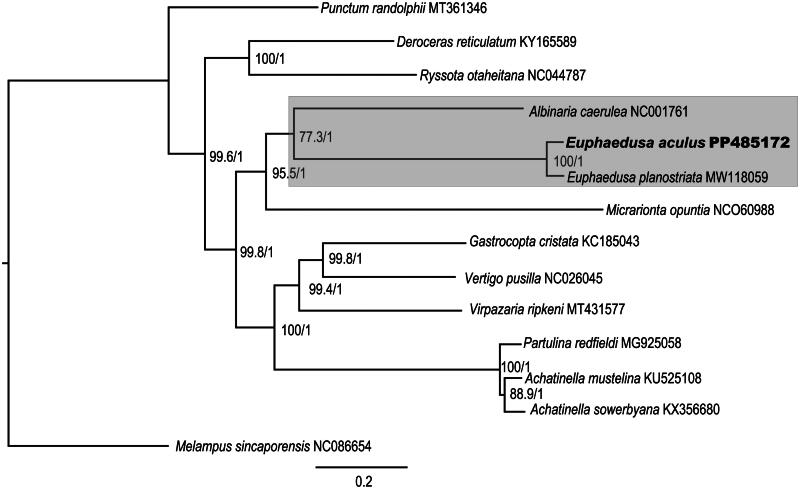
Phylogenetic trees generated using maximum likelihood and Bayesian interference based on the 13 protein-coding genes and two rRNA genes. ML bootstrap and BI posterior probability values were shown on the nodes. The mitogenome sequences used including *Deroceras reticulatum* KY765589 (Ahn et al. 2017); *Ryssota otaheitana* NC044784 (Guzmán et al. 2021); *Punctum randolphii* MT361346; *Albinaria caerulea* NC001761; *Euphaedusa planostriata* MW118059 (Zhang et al. 2021); *Euphaedusa aculus* PP485172 (this study); *Micrarionta opuntia* NC060988; *Gastrocopta cristata* KC185403 (Groenenberg et al. 2017); *Vertigo pusilla* NC026045 (Schultz et al. 2018); *Virpazaria ripkeni* MT431577; *Achatinella mustelina* KU525108, *Achatinella sowerbyana* KX356680 (Price et al. 2016); *Partulina redfieldi* MG925057 (Price et al. 2018); *Melampus sincaporensis* NC086654.

The ML tree and the BI tree demonstrated congruent evolutionary relationships, They both revealed that the *E. aculus* sequenced in this study is sister to *E. planostriat* and then clustered with *Albinaria caerulea*, which showed Clausiliidae a monophyly (Figure 3). The species *Micrarionta opuntia* from the family Helminthoglyptidae is a sister clade to the clausiliids (Figure 3).

## 4. Discussion and conclusion

The door snails from Clausiliidae have an extensive species diversity and breeding diversity. However, there are only two species (i.e. *Albinaria caerulea* and *Euphaedusa planostriata*) with complete mitogenome published in GenBank until this present study. In this study, we assembled the nearly complete mitogenome of *E. aculus*, which represented the second mitogenome of species from the genus *Euphaedusa*. Together with 12 other stylommatophora species, we reconstructed the phylogenetic trees and revealed a clear phylogenetic relationship among the genus of the family Clausiliidae. Uit de Weerd and Gittenberger (2013) divided 67 Clausilidae species into seven subfamilies by the phylogenetic analysis of 28S rRNA and histone H3 and H4. The genus *Albinaria* and *Euphaedusa* used in this study belonged to the clade Phaedusinae +Serrulininae and Alopiinae as proposed by Uit de Weerd and Gittenberger (2013), respectively. The phylogenetic results from this study revealed a sister clade of *Euphaedusa* to *Albinaria*, which was consistent with subfamily level phylogenetic relationships from Uit de Weerd and Gittenberger (2013). Our study provided important information for further studying species identification, population genetics, and phylogenetic relationships among the door snails.

## Supplementary Material

Supplementary Figure 1.jpg

## Data Availability

The genome sequence data that support the findings of this study are openly available in GenBank of NCBI at https://www.ncbi.nlm.nih.gov under the accession no. PP485172. The associated BioProject, SRA, and Bio-Sample numbers are PRJNA1086373, SRX23896143, and SAMN40374704, respectively.
