# Supplementary figures and images for: The nearly complete mitochondrial genome of the door snail Euphaedusa aculus (Stylommatophora: Clausiliidae) and phylogenetic analysis
Source: Mitochondrial DNA B Resour. 2024 Nov 20;9(11):1581–5. doi: 10.1080/23802359.2024.2427105 (PMC11580148; doi:10.1080/23802359.2024.2427105)

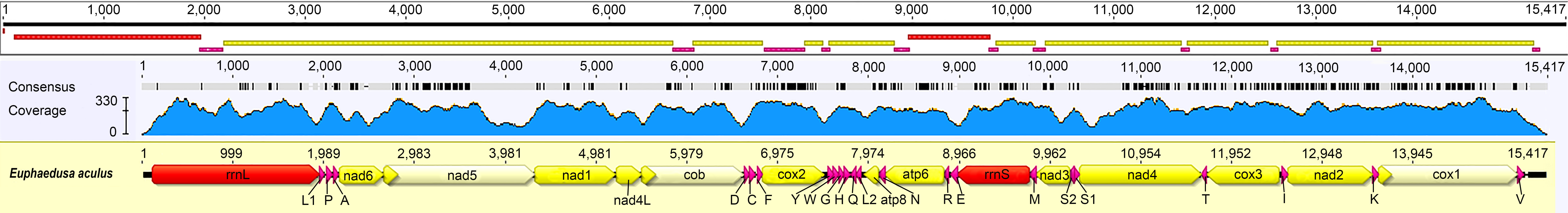

Supplement: Supplementary Figure 1.jpg [file TMDN_A_2427105_SM2392.jpg]
